# Patellar mobility can be reproducibly measured using ultrasound

**DOI:** 10.1007/s10195-014-0301-3

**Published:** 2014-06-04

**Authors:** Takashi Kanamoto, Yoshinari Tanaka, Yasukazu Yonetani, Keisuke Kita, Hiroshi Amano, Masashi Kusano, Mie Fukamatsu, Shinji Hirabayashi, Shuji Horibe

**Affiliations:** 1 Department of Rehabilitation, Osaka Rosai Hospital, 1179-3 Nagasone-cho, Kita-ku, Sakai, Osaka 597-8025 Japan; 2 Department of Orthopaedic Surgery, Osaka Rosai Hospital, 1179-3 Nagasone-cho, Kita-ku, Sakai, Osaka 597-8025 Japan; 3 Department of Clinical Laboratory, Osaka Rosai Hospital, 1179-3 Nagasone-cho, Kita-ku, Sakai, Osaka 597-8025 Japan

**Keywords:** Patellar mobility, Ultrasound, Rehabilitation, Isometric knee extension exercise, Reliability

## Abstract

The present study was performed to examine the reliability of ultrasound in evaluating patellar mobility in the superior–inferior direction. Twelve healthy men volunteered for the study. Patellar mobility in the superior–inferior direction during isometric knee extension contraction with the knee immobilized in a semi-flexed knee brace was measured using ultrasound. Both intra-observer and inter-observer reliability were assessed by intra-class correlation coefficients (ICCs). Bland–Altman analysis was used for assessing agreement between measurements. ICC values were excellent for both intra-observer and inter-observer reliability at 0.97 and 0.93, respectively. In 95 % of measurements, the same observer measured within −0.55 to 0.61 mm, while different observers measured within −0.82 to 0.85 mm. In conclusion, patellar mobility in the superior–inferior direction during an isometric knee extension exercise can be reproducibly measured using ultrasound.

VI (basic study of a novel evaluation method).

## Introduction

To prevent postoperative complications, it is important to regain normal patellar mobility after knee surgeries such as anterior cruciate ligament reconstruction and total knee arthroplasty. Patellar immobility leads to decreased range of motion, quadriceps inhibition, altered gait pattern, and prolonged rehabilitation [1]. Thus, multidirectional mobilization of the patella and quadriceps muscle setting exercises are initiated in the early postoperative period to improve patellar mobility and quadriceps function. Despite the importance of this being generally accepted, a single gold standard evaluation method of patellar mobility has not been established [2–4].

With the recent development of high-resolution probes, the use of musculoskeletal ultrasound has significantly increased [5]. The superficial localization of the knee extensor apparatus, including the patella and patellar tendon, makes it suitable for ultrasound evaluation [6, 7]. The purpose of this report was to determine if ultrasonography can be useful in evaluating patellar mobility in the superior–inferior direction during an isometric knee extension exercise.

## Materials and methods

### Participants

Twelve healthy men with no signs of musculoskeletal injury or disorder that would prevent their participation volunteered for the study. Mean (±SD) age, height, and weight were 31.2 ± 6.9 years, 175 ± 3.7 cm, and 67.8 ± 8.6 kg, respectively. Ultrasound examinations were performed by an orthopedic surgeon and an ultrasonographer experienced in musculoskeletal ultrasound measurements.

### Procedure

**Fig. 1:**
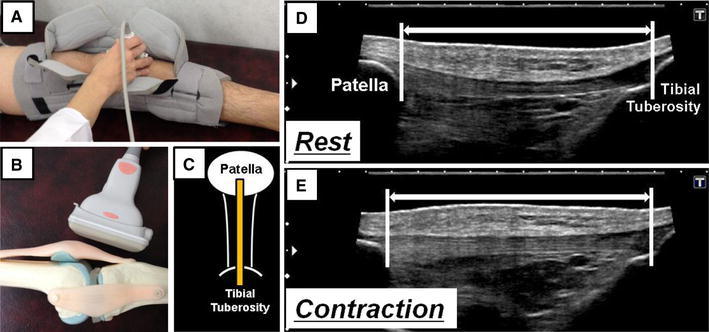
Ultrasound examination of the patellar mobility in the superior–inferior direction during isometric knee extension exercise. **a** The participants were evaluated in a supine position with the knee immobilized with a semi-flexed brace. **b** An ultrasound probe was fitted onto the skin overlying the patellar tendon in the sagittal plane using water bag kit. **c** The probe was positioned so that the caudal pole of the patella and the tibia tuberosity were visible within the viewing field. **d**, **e** Ultrasound images of the patella, patellar tendon, and tibial tuberosity at rest and during isometric knee extension contraction. The patellar mobility in the superior–inferior direction was measured as the change in distance between manually marked points of the deep insertion to the caudal pole of the patella and tibia tuberosity

Participants performed three trials of maximal knee extension contractions in the supine position with the knee immobilized by a semi-flexed knee brace (Fig. 1a). An 8.0 MHz, 58-mm ultrasound probe (Aplio™ 300, Toshiba, Tokyo, Japan) was fitted onto the skin overlying the patellar tendon in the sagittal plane using a water bag kit (UAWB-022A, Toshiba). The probe was positioned so that the inferior pole of the patella and the tibial tuberosity were within the viewing field during isometric knee extension contraction (Fig. 1b–e).

All trials were performed on two different days and analyzed independently by two observers. Using recorded ultrasound images, the observer manually marked the points of the inferior pole of the patella and the tibial tuberosity frame by frame. The patellar mobility in the superior–inferior direction was defined as the change in distance between the two points during isometric knee extension contraction.

### Statistical analysis

Continuous variables are expressed as mean ± SD. Both intra-observer and inter-observer reliability were assessed using intra-class correlation coefficients (ICC). Bland–Altman analysis was used to assess agreement between measurements. All statistical analyses were performed using SPSS (SPSS Inc., Chicago, IL, USA).

## Results

The intra-observer reproducibility for patellar mobility was excellent, with an ICC (1, 3) of 0.97. In Bland–Altman plots, the mean difference between paired measurements by two observers was 0.03 mm. The corresponding 95 % limits of agreement were −0.55 to 0.61 mm (Table 1).

**Table 1:** .

	Mean ± SD		ICC (95 % CI)	Mean difference	Limits of agreement
Intra-observer	Day 1	Day 2			
Patella–tuberosity distance (mm)					
Rest	44.2 ± 5.3	44.4 ± 5.2	0.99 (0.96–1.00)	−0.22	−1.88 to 1.43
Contraction	47.3 ± 5.4	47.5 ± 4.7	0.98 (0.94–1.00)	−0.2	−2.1 to 1.8
Patellar mobility (mm)	3.1 ± 1.2	3.1 ± 1.2	0.97 (0.91–0.99)	0.03	−0.55 to 0.61

	Mean ± SD		ICC (95 % CI)	Mean difference	Limits of agreement
Interobserver	Observer 1	Observer 2			
Patella–tuberosity distance (mm)					
Rest	44.2 ± 5.3	44.0 ± 5.2	0.98 (0.93–0.99)	0.2	−2.2 to 2.5
Contraction	47.3 ± 5.4	47.1 ± 5.4	0.98 (0.94–0.99)	0.2	−2.0 to 2.3
Patellar mobility (mm)	3.1 ± 1.2	3.1 ± 0.9	0.93 (0.77–0.98)	0.02	−0.82 to 0.85

The inter-observer reproducibility for patellar mobility was also excellent, with an ICC (2, 3) of 0.93. In Bland–Altman plots, the mean difference between paired measurements by two observers was 0.02 mm. The corresponding 95 % limits of agreement were −0.82 to 0.85 mm (Table 1).

## Discussion

The principal findings of the present study were that patellar mobility in the superior–inferior direction during an isometric knee extension exercise could be reproducibly measured using ultrasound. ICC values were excellent for both intra-observer and inter-observer reliability at 0.97 and 0.93, respectively. In 95 % of measurements, the same observer measured within −0.55 to 0.61 mm, while different observers measured within −0.82 to 0.85 mm.

Ultrasound evaluation of the patellar tendon has been used extensively in recent years. There are several publications describing the ultrasound appearance of patellar tendinopathy and ultrasound measurements of mechanical properties of the patellar tendon [6, 7]. However, to our knowledge, this is the first report showing the utility of ultrasound in evaluating patellar mobility during a knee rehabilitation exercise.

Measurement of patellar tendon length with ultrasound using adjustable surface markers and calipers is highly accurate and has good inter-observer reliability [8]. Hansen et al. [6] performed patellar tendon measurements, keeping 90° of flexion with a custom made rigid cast to position an ultrasound probe, and showed high accuracy and reproducibility using measurements from two trials after discarding trials with the smallest and largest measurements. In a recent report, Schulze et al. [9] concluded that 5–6 trials are required for reliably measuring tendon elongation. In the present study, simple tools available in the clinical setting were used to perform the measurements, and an average of three trials showed sufficient reliability for clinical application.

Although abnormal patellar mobility potentially contributes to several knee disorders, such as anterior knee pain, patellofemoral pain, and arthrofibrosis, there is no strong evidence to support its importance, partially due to the lack of a standard measurement method [1, 3, 4, 10]. Considering the patient tolerability, low cost, and lower time commitment of our simple method, clinicians can easily evaluate patellar mobility before and after treatment. In addition to the conventional assessment of quadriceps muscle strength, this method will help establish appropriate and effective treatment strategies [1]. Furthermore, objective evaluation of patellar mobility in the clinical context has the potential to provide clues to underlying causes of knee disorders as well as monitor treatment effects.

Limitations of the current study include the small sample size and the fact that the general condition of participants was not assessed. Although ICCs of intra-observer and inter-observer reproducibility were high, further study is required to clarify the utility of the present method for a large cohort. Another limitation is that we did not include measures of quadriceps muscle force. In the future, it would also be interesting to test the relationship between muscle force and patellar mobility.

In conclusion, patellar mobility in the superior–inferior direction during an isometric knee extension exercise can be reproducibly measured using ultrasound. Clinical application should provide useful information for treatment evaluation and planning in rehabilitation therapy.
